# Trends and development in perioperative enteral nutrition: a systematic bibliometric analysis

**DOI:** 10.3389/fnut.2024.1406129

**Published:** 2024-09-13

**Authors:** Chen Luo, Jianing Yin, Yuejiao Sha, Wei Gong, Ling Shen

**Affiliations:** ^1^Department of Nursing, Xinhua Hospital, Shanghai Jiao Tong University School of Medicine, Shanghai, China; ^2^School of Nursing, Shanghai Jiao Tong University, Shanghai, China; ^3^Department of Pediatric Critical Care Medicine, Xinhua Hospital, Shanghai Jiao Tong University School of Medicine, Shanghai, China; ^4^Department of General Surgery, Xinhua Hospital, Shanghai Jiao Tong University School of Medicine, Shanghai, China; ^5^Shanghai Key Laboratory of Biliary Tract Disease Research, Research Institute of Biliary Tract Disease, Shanghai Research Center of Tract Disease, Shanghai Jiao Tong University School of Medicine, Shanghai, China

**Keywords:** perioperative, enteral nutrition, research trends, hotspots, bibliometric analysis

## Abstract

**Background:**

This research aims to explore the intellectual landscape of studies in perioperative enteral nutrition (PEN) and identify trends and research frontiers in the field.

**Methods:**

Scientometric research was conducted through the analysis of bibliographic records from the Web of Science Core Collection Database for the period 2014–2023. Analyses performed using CiteSpace software included cooperation network analysis, reference co-citation analysis, and keywords co-occurrence analysis.

**Results:**

The analysis included 3,671 valid records in the final dataset. Findings indicate an upward trend in annual publications, with the United States leading in research output and Harvard University as the top publishing institution. The Journal of Parenteral and Enteral Nutrition was identified as the most productive journal. Notable research hotspots include enhanced recovery after surgery, early enteral nutrition, intestinal failure, short bowel syndrome, abdominal surgery. Evidence-based articles have emerged as the predominant literature type. Future research trends are anticipated to focus on gut microbiota and patients with congenital heart disease.

**Conclusion:**

Our study provides a comprehensive analysis of the publication volume, contributions by country/region and institutions, journal outlets, and reference and keyword clusters in the field of PEN over the decade. The findings provide valuable insights for researchers, policymakers, and clinicians, helping them comprehend the research landscape, identify gaps, and shape future research directions in this field.

## 1 Introduction

Enteral nutrition (EN) supports or restores nutritional balance through oral nutritional supplements (ONS) or tube feeding (TF) (1). ONS is appropriate for patients at high risk of malnutrition or those unable to meet their nutritional needs through regular oral intake. Conversely, TF is necessary for patients who are unable to consume food orally or whose oral intake does not satisfy their nutritional needs (2). TF involves delivering gastrointestinal nutrition via a catheter or stoma, typically via nasogastric tubes, gastric stomas, or jejunal stomas (3). The advantages of EN include maintaining intestinal mucosal integrity and barrier function, enhancing nutrient absorption, regulating intestinal microecology, preventing intestinal flora translocation and protecting gut immune function (4).

Perioperative nutritional intervention (PEN) encompasses both preoperative and postoperative stages. Preoperative supplementation prepares the body for the metabolic stress of surgery, proving especially beneficial for severely malnourished patients. Early enteral nutrition (EEN) has been demonstrated to enhance wound healing, reduce the rate of postoperative infections, and decrease the incidence of complications such as anastomotic fistula, delayed gastric emptying, and recurrent laryngeal nerve palsy (5). Although the importance of PEN cannot be overstated, it also presents risk, including a 58.5% incidence of enteral nutrition feeding intolerance (ENFI) among critically ill patients (6). Patients with gastrointestinal tumors often experience nausea and diarrhea during ONS, leading to reduced or halted EN (7). Furthermore, a multicenter randomized controlled trial indicated that EN could cause intestinal ischemia and acute pseudo-obstruction of the colon in critically ill patients on mechanical ventilation and vasoactive drugs (8).

As research in PEN progresses, a primary challenge for newcomers is to swiftly identify emerging topics and innovations. Bibliometric analysis is a crucial tool for objectively assessing research trends, hotspots, and recent advancements (9). To date, limited bibliometric studies have focused on the developmental trends in PEN. Therefore, this article employs bibliometric analysis to review the Web of Science Core Collection (WoSCC) Database related to PEN. It examines yearly publication volumes, geographic distribution, institutional contributions, references, journal sources, and keyword citations, delving into the analysis of current research hotspots and cutting-edge knowledge. This paper aims to serve as a resource for clinical practitioners and medical researchers.

## 2 Methods

### 2.1 Dataset

Data from the Web of Science Core Collection Database was selected for its high quality and rigorous peer-review process, ensuring the credibility and accuracy of the information. The Web of Science also provides detailed citation information and advanced analysis tools, invaluable for conducting citation analysis and network analysis (8). This study utilized CiteSpace 6.3.1 Basic (10), developed by Chen’s team, for the bibliometric analysis and visualization of the literature.

Regarding data collection, the following retrieval strategy was developed: “TS = [(enteral nutrition or enteral feeding or intestinal nutrition or intestinal feeding) and (perioperative or preoperative or postoperative or surgery or surgical)]. “(Table 1) The language was set to “English”; literature category to “Article” and “Review Article”; time frame from 1 January 2014 to 31 December 2023. Exclusions included early access, retracted publication, proceeding paper, book chapter, publication with expression of concern.

**Table 1:** Search process and results in the Web of Science Core Collection Database.

Number	Search strategy	Retrieval Result
#1	TS = (enteral nutrition) OR (enteral feeding) OR (intestinal nutrition) OR (intestinal feeding)	71,674
#2	TS = perioperative OR postoperative OR preoperative OR surgery OR surgical	2,423,473
#3	#1 and #2	8,245
#4	#3 and Article or Review Article (Document Types)	7,862
#5	#4 and 2023 or 2022 or 2021 or 2020 or 2019 or 2018 or 2017 or 2016 or 2015 or 2014 (Publication Years)	3,916
#6	#5 and English (Languages)	3,786
#7	Remove duplications from #6	3,671

**Figure 1:**
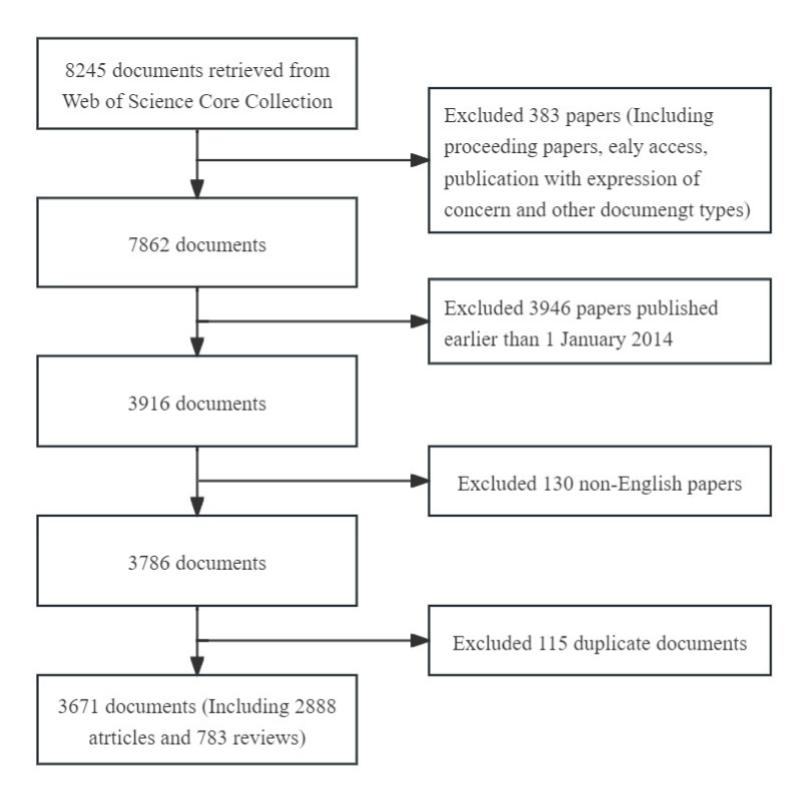
A frame flow diagram showing the detailed selection criteria and bibliometric analysis steps of PEN.

Using this search criteria, 3,786 pieces of literature were identified. The “Full Record and Cited References” of these records were extracted in “Plain text” format into CiteSpace software. Hundred and fifteen duplicate records were identified using the software’s native function of checking duplicates. Consequently, 3,671 literature papers comprised the final dataset. The frame flow diagram of the literature selection process is shown in Figure 1.

### 2.2 Software and parameter setting

CiteSpace is an indispensable tool for uncovering hidden insights in scientific literature through visual bibliometric analyses. It generates “scientific knowledge maps” that illustrate the structure and distribution patterns of scientific knowledge. These maps include institutional and national collaboration maps, reference co-citation cluster maps, burst maps, keyword co-occurrence maps, clustering maps, timeline maps, and burst maps (11).

Upon importing data into CiteSpace, the analysis period was set from January 2014 to December 2023, utilizing annual time slices with network connections determined by the “Cosine” method, and limited to “Within slices.” For each slice, the top 50 entries with the most citations or occurrences were selected, with Term Source including Title, Abstract, Author Keywords, and Keywords Plus. The analysis in CiteSpace involved:

Annual publication volume: Using Microsoft Office Excel 2016 and Origin 2024 to compute the annual publication volume and generate a trend chart.

Publications by country/region: Choosing only “Country” in Node Types, with a scale factor *k* = 25, for the time slicing from January 2014 to December 2023.

Publications by institution: Choosing only “Institution” in Node Type, with a scale factor *k* = 15, for the time slicing from January 2014 to December 2023.

Journal analysis: Choosing only “Cited Journal” in Node Types, with a scale factor *k* = 15, for the time slicing from January 2014 to December 2023.

Keyword analysis: Choosing only “Keyword” in Node Types, with a scale factor *k* = 5, for the time slicing from January 2014 to December 2023.

Reference analysis: Choosing only “Reference” in Node Types, with a scale factor *k* = 5, for the time slicing from January 2014 to December 2023.

All maps underwent pruning using Pathfinder and Pruning the merged network.

Both reference co-citation clustering and keyword clustering analysis, focused on “Keywords” using the Log-Likelihood Ratio (LLR) algorithm. The resulting maps display Modularity clustering module values (*Q* values) and average Silhouette values (*S* values) in the top left corner, which help evaluate the clustering quality. A *Q* value falls within the [0, 1] range. A *Q* value greater than 0.3 signifies significant clustering structure, above 0.5 suggests reasonable clustering, and values exceeding 0.7 indicate highly convincing clustering results.

For keyword burst map and reference burst map, the γ value [0,1] is set to 1.0, and the Minimum Duration is adjusted to 1 year.

Keyword co-occurrence maps, clustering maps, timeline maps, and burst maps are all based on the co-occurrence of keywords in the cited literature, providing comprehensive insights into keyword frequency, centrality, clustering structure, cluster time spans, and the progression of research themes and keyword evolution (12). Timeline maps convert the co-occurrence map into a timeline format and display a legend label for every year.

## 3 Results

### 3.1 General information

In the Web of Science Core Collection, we identified 3,786 pertinent documents, involving 22,386 authors, 13,355 research institutions, and originating from 573 countries or regions, published across 968 journals.

### 3.2 Annual publication volume

**Figure 2:**
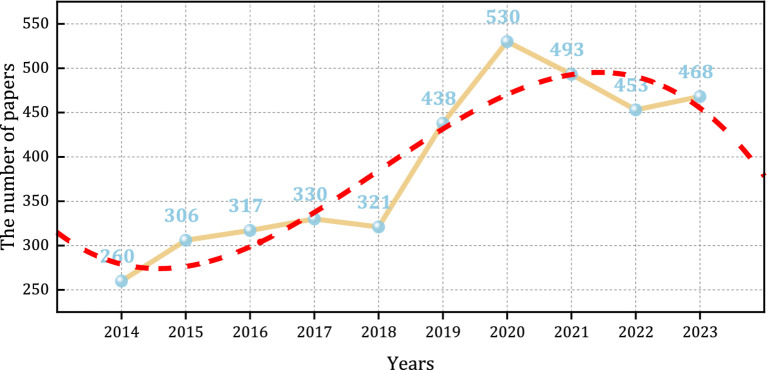
Trend of publication outputs from 2014 to 2023 on PEN.

This paper analyzes data from articles published over the last decade. The rising number of publications in the PEN suggests an increasing scholarly interest in this field, especially during the period from 2018 to 2020, which saw rapid growth, peaking at 530 publications in 2020. There was a slight decrease in publication volume between 2020 and 2022 (Figure 2). Polynomial fitting of the yearly cumulative publication volume, resulting in the equation $y=-1.38x^{3}+833.08x^{2}-1.68\times 10^{7}x+1.13\times 10^{10}$, with a fitting goodness of R^2^ = 0.86511, indicating a good fit.

### 3.3 Countries/regions analysis

**Figure 3:**
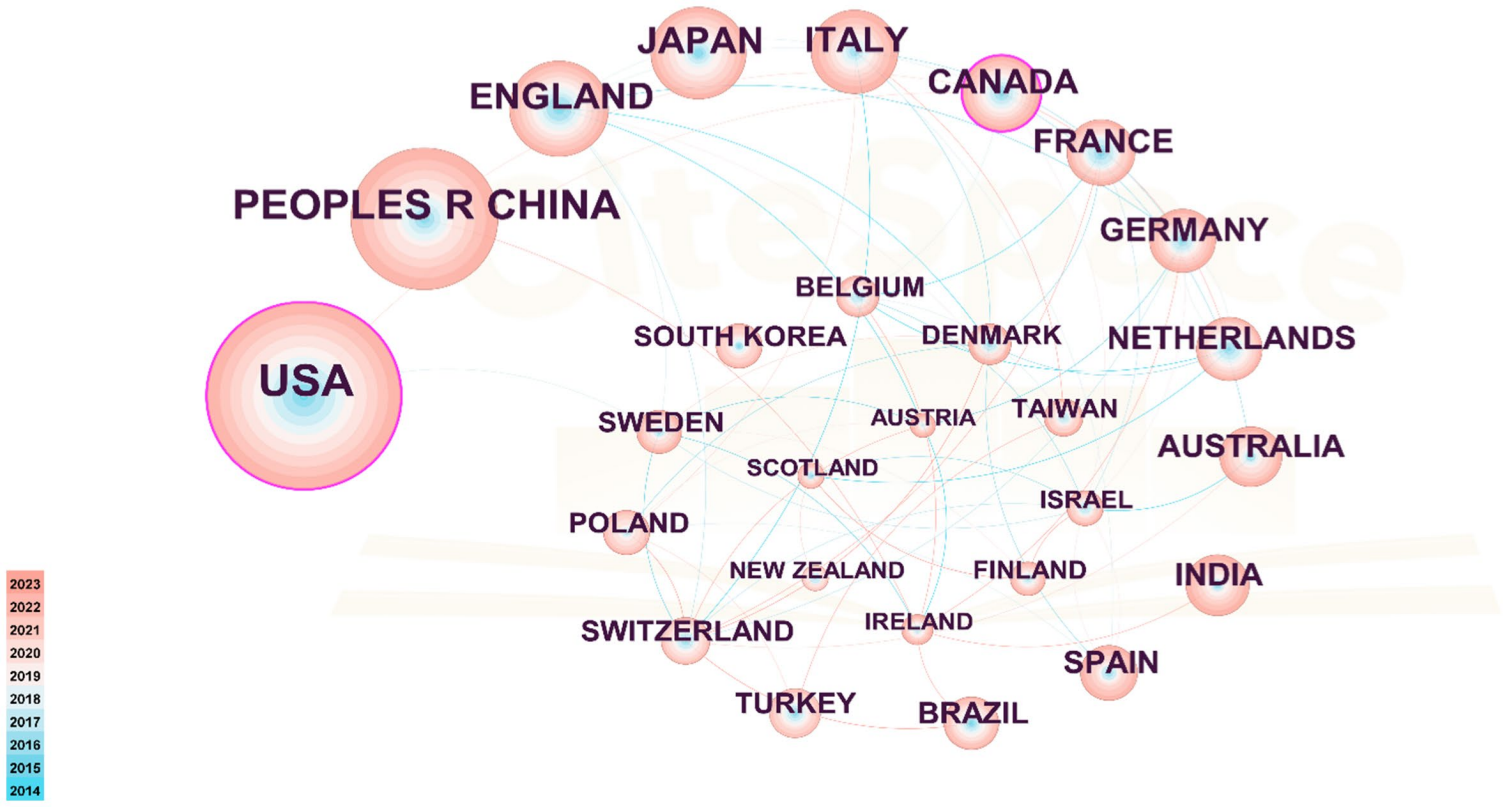
Cooperation network among countries/regions. The nodes in the graph represent countries/regions, and the lines between the nodes represent the collaborative relationships. The color depth of lines between two countries/regions indicates the strength of cooperation. The bigger the node, the higher volume of original research.

Table 2 presents the distribution of PEN research publications by country/region, highlighting the United States as the leader with 1,044 publications, followed by PEOPLES R China with 640 publications. These two countries far outpace others in publication volume. Additionally, England, Japan, Italy, and other countries each have contributed over 200 publications. In terms of centrality, Canada ranks first with a score of 0.12, followed by USA (0.11), Israel (0.09), England (0.08), France (0.08) and India (0.08). Figure 3 illustrates the collaboration map among countries/regions, featuring 122 nodes and 328 links with a density of 0.0444, highlighting the USA, the PEOPLES R CHINA and ENGLAND as active contributors. Despite high publication volumes, China and Japan show limited international collaborations.

**Table 2:** Top 10 countries/regions in the PEN study.

Ranking	Country	Count	Centrality
1	USA	1,044	0.11
2	PEOPLES R CHINA	640	0.00
3	ENGLAND	287	0.08
4	JAPAN	280	0.00
5	ITALY	233	0.05
6	CANADA	185	0.12
7	FRANCE	149	0.08
8	NETHERLANDS	145	0.03
9	GERMANY	138	0.05
10	AUSTRALIA	124	0.05

### 3.4 Institution analysis

**Figure 4:**
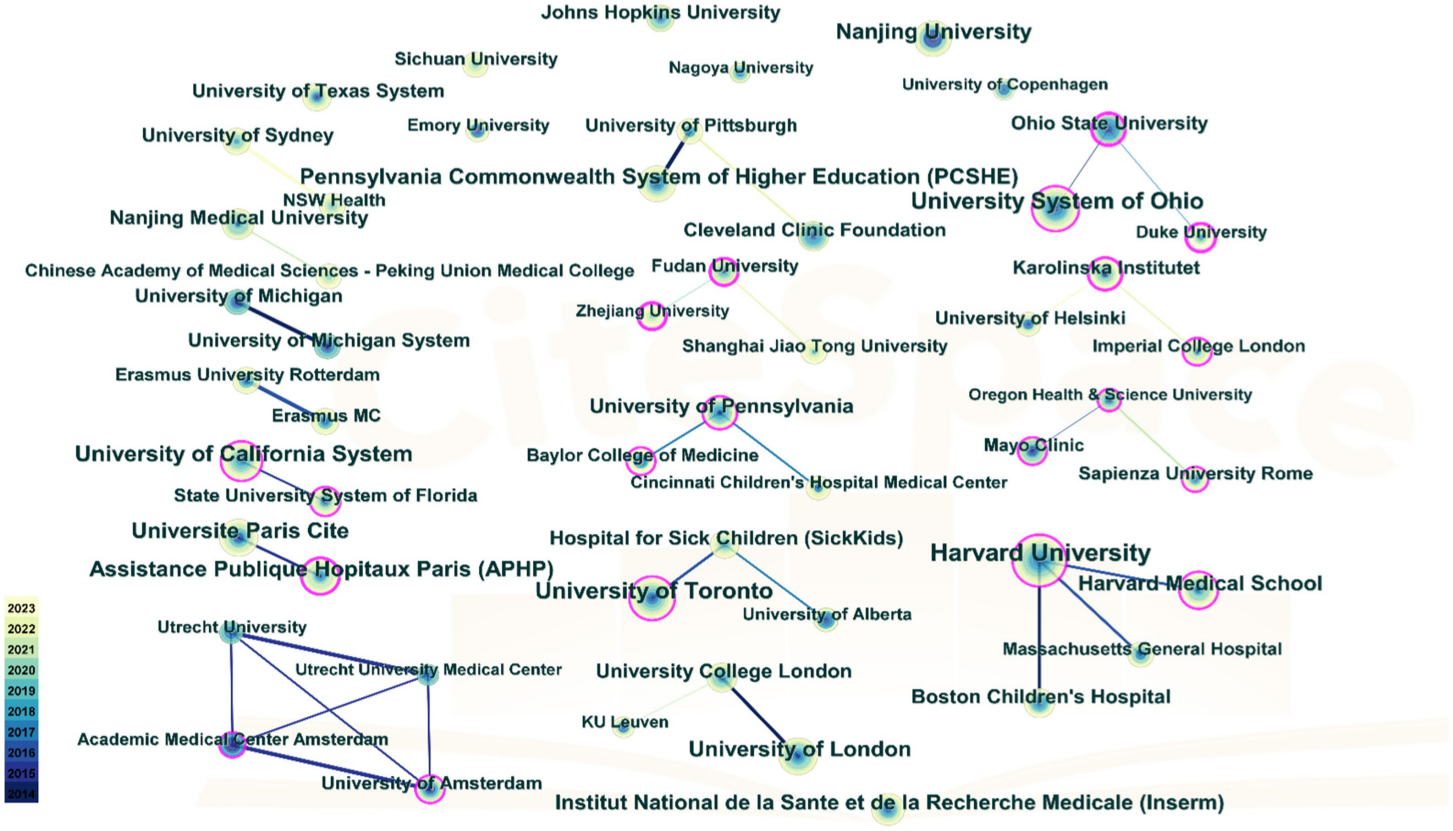
Cooperation network among institutions. The nodes in the graph represent institutions, and the lines between the nodes represent the collaborative relationships. The color depth of lines between two institutions indicates the strength of cooperation. The bigger the node, the higher volume of original research.

Table 3 details institutional publications, indicating that universities predominantly lead in publication volume. Harvard University ranks first with a remarkable 109 publications, followed by University System of Ohio with 86 publications, University of Toronto with 80 publications, and University of California System with 71 publications, underscoring the significant contributions of North American institutions on PEN. Figure 4 depicts the institutional cooperation network map, featuring 269 nodes and 1709 links, with a density of 0.0474. Notably, Ohio State University exhibits the highest centrality (0.49), having collaborated closely in recent years with Duke University and the University System of Ohio.

**Table 3:** Top 10 institutions in the PEN study.

Ranking	Institution	Count	Centrality
1	Harvard University	109	0.03
2	University System of Ohio	86	0.04
3	University of Toronto	80	0.13
4	University of California System	71	0.10
5	Assistance Publique Hopitaux Paris (APHP)	66	0.08
6	Universite Paris Cite	63	0.08
7	Nanjing University	61	0.02
8	Pennsylvania Commonwealth System of Higher Education (PCSHE)	60	0.04
9	University of London	59	0.05
10	Harvard Medical School	55	0.02

### 3.5 Reference analysis

**Figure 5:**
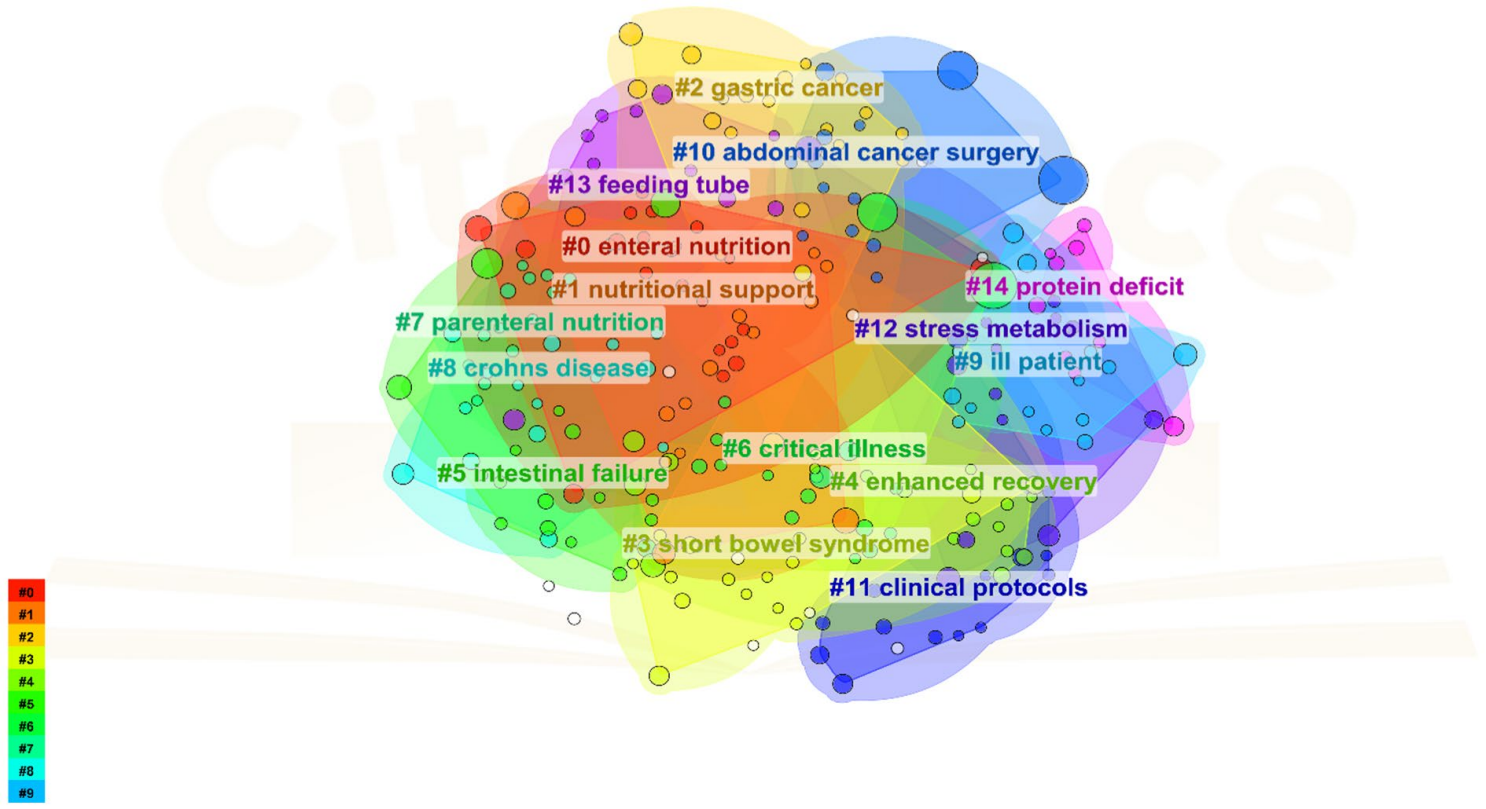
Cited cluster analysis showing the 14 main research areas.

The knowledge base of a research field comprises its collection of references, with the citing literature reflecting the research frontiers. Using CiteSpace, a cluster analysis was conducted on references concerning PEN. The graph features 238 nodes and 263 links, with a density of 0.0093. The referenced literature is categorized into 15 clusters, namely, #0 Enteral nutrition, #1 Nutritional support, #2 Gastric cancer, #3 Short bowel syndrome, #4 enhanced recovery, #5 Intestinal failure, #6 critical illness, #7 Parenteral nutrition, #8 Crohn’s disease, #9 Ill patient, #10 Abdominal cancer surgery, #11 Clinical protocols, #12 Stress metabolism, #13 Feeding tube, and #14 Protein deficit (Figure 5).

**Figure 6:**
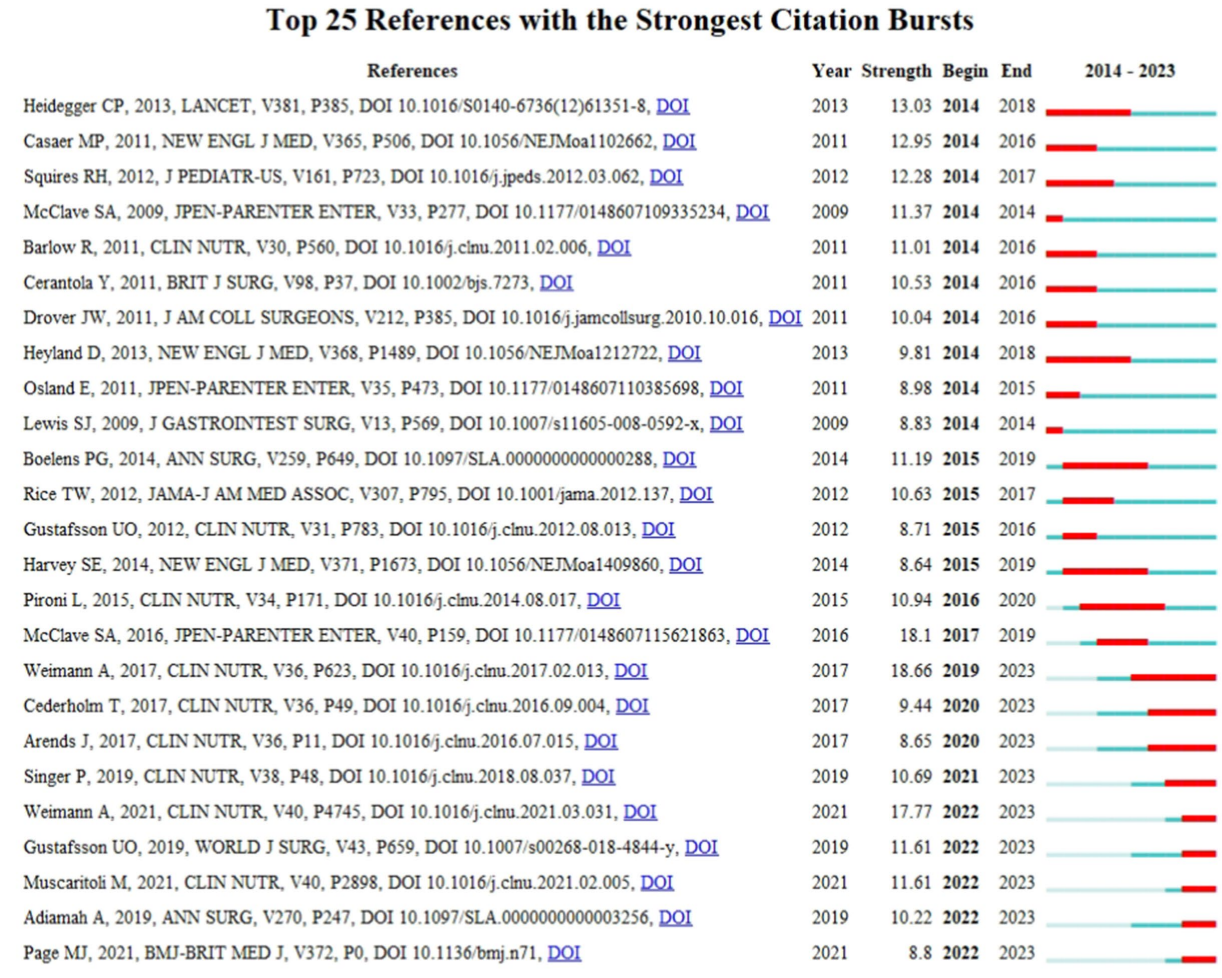
Top 25 references with the strongest citation bursts.

Figure 6 illustrates the top 25 references with the most significant co-citation bursts. We obtained 9 references burst in recent years, including “ESPEN guideline: Clinical nutrition in surgery” by Weimann et al. (13) (2019–2023), “ESPEN guidelines on definitions and terminology of clinical nutrition” by Cederholm et al. (14) (2020–2023), “ESPEN guidelines on nutrition in cancer patients” by Arend et al. (15) (2020–2023), “The European society for parenteral and enteral nutrition (ESPEN) guideline on clinical nutrition in the intensive care unit(ICU)” by Singer et al. (16) (2021–2023), “ESPEN practical guideline: Clinical nutrition in surgery” by Weimann et al. (17) (2022–2023), “Guidelines for Perioperative Care in Elective Colorectal Surgery: Enhanced Recovery After Surgery (ERAS®) Society Recommendations:2018” by Gustafsson et al. (18) (2022–2023), “ESPEN Practical guideline: Clinical Nutrition in cancer” by Muscaritolo et al. (1) (2022–2023), “Response to the Comment on ‘The Impact of Preoperative Immune-Modulating Nutrition on Outcomes in Patients Undergoing Surgery for Gastrointestinal Cancer’: A Systematic Review and Meta-analysis” by Adiamah et al. (19) (2022–2023), and “The PRISMA 2020 statement: an updated guideline for reporting systematic reviews” by Page et al. (20) (2022–2023). These articles, underscoring the importance of evidence-based practice, enable researchers and clinicians to access the latest and most comprehensive medical evidence, thus enhancing patient care in a scientifically rigorous manner.

### 3.6 Journal analysis

A total of 968 journals have published papers related to the PEN. Table 4 presents the top 10 journals ranked by publication volume in this field. The Journal of Parenteral and Enteral Nutrition leads with 130 papers, following by the Journal of Pediatric Surgery with 107 papers, Nutrients with 103 papers, and Nutrition in Clinical Practice with 102 papers. Notably, three of these journals are classified as JCR Q1 journals.

**Table 4:** Top 10 journals ranked by number of publications in the PEN.

Ranking	Journal	Documents	JCR (2023)	IF (2023)
1	Journal of Parenteral and Enteral Nutrition	130	Q2	3.2
2	Journal of Pediatric Surgery	107	Q1	2.4
3	Nutrients	103	Q1	4.8
4	Nutrition in Clinical Practice	102	Q3	2.1
5	Clinical Nutrition	90	Q1	6.6
6	Medicine	62	Q2	1.3
7	Pediatric Surgery International	61	Q2	1.5
8	Journal of Pediatric Gastroenterology and Nutrition	48	Q3	2.4
9	Nutrition	42	Q2	3.2
10	Clinical Nutrition ESPEN	41	Q3	2.9

### 3.7 Analysis of keywords

#### 3.7.1 High-frequency keywords and co-occurrence

**Figure 7:**
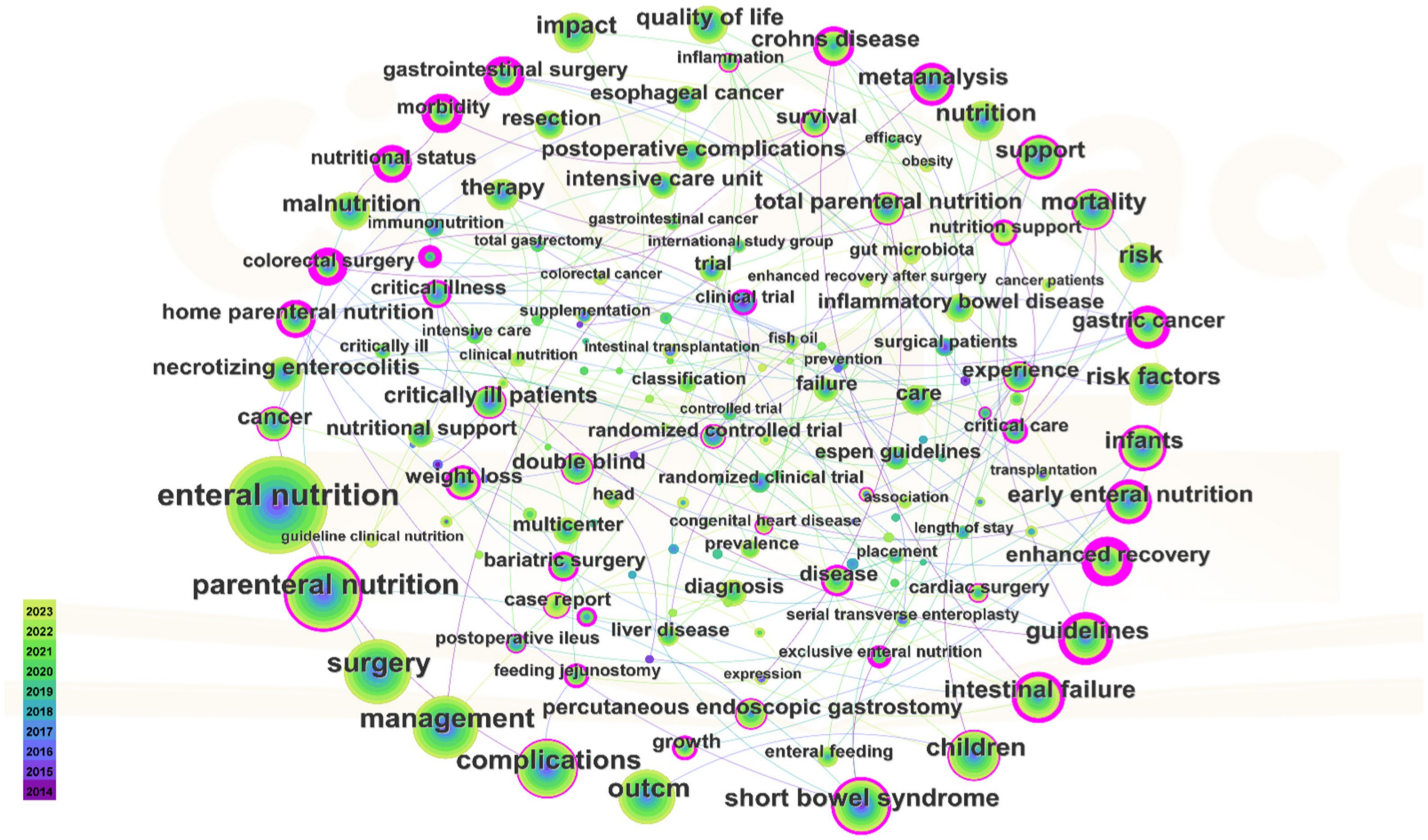
Keywords with highly frequency. The bigger the keywords, the more frequently they appear in the articles.

Figure 7 presents a co-occurrence graph for keywords appearing at least 50 times. The graph features 154 nodes and 178 links, with a density of 0.0151. In the graph, keyword size corresponds to its frequency in journal occurrences. Table 5 reveals that the most frequently occurring keyword is “Enteral nutrition,” followed by “Parenteral nutrition.” The keywords with high centrality are “Early enteral nutrition,” “Mortality,” “Short bowel syndrome,” “Children” and “Intestinal failure,” with “Guideline” being a prevalent literature type.

**Table 5:** Top 20 keywords cited articles in the PEN study.

Ranking	Keyword	Count	Centrality	Ranking	Keyword	Count	Centrality
1	Enteral nutrition	1,033	0.13	11	Intestinal failure	236	0.39
2	Parenteral nutrition	573	0.10	12	Guidelines	227	0.54
3	Surgery	511	0.00	13	Nutrition	208	0.05
4	Management	457	0.03	14	Mortality	204	0.43
5	Complications	379	0.13	15	Quality of life	195	0.03
6	Outcome	368	0.00	16	Support	192	0.11
7	Children	289	0.39	17	Infants	191	0.10
8	Short bowel syndrome	272	0.42	18	Malnutrition	188	0.00
9	Impact	237	0.03	19	Early enteral nutrition	179	0.45
10	Risk factors	236	0.00	20	Meta-analysis	172	0.54

#### 3.7.2 Analysis of keyword clustering

**Figure 8:**
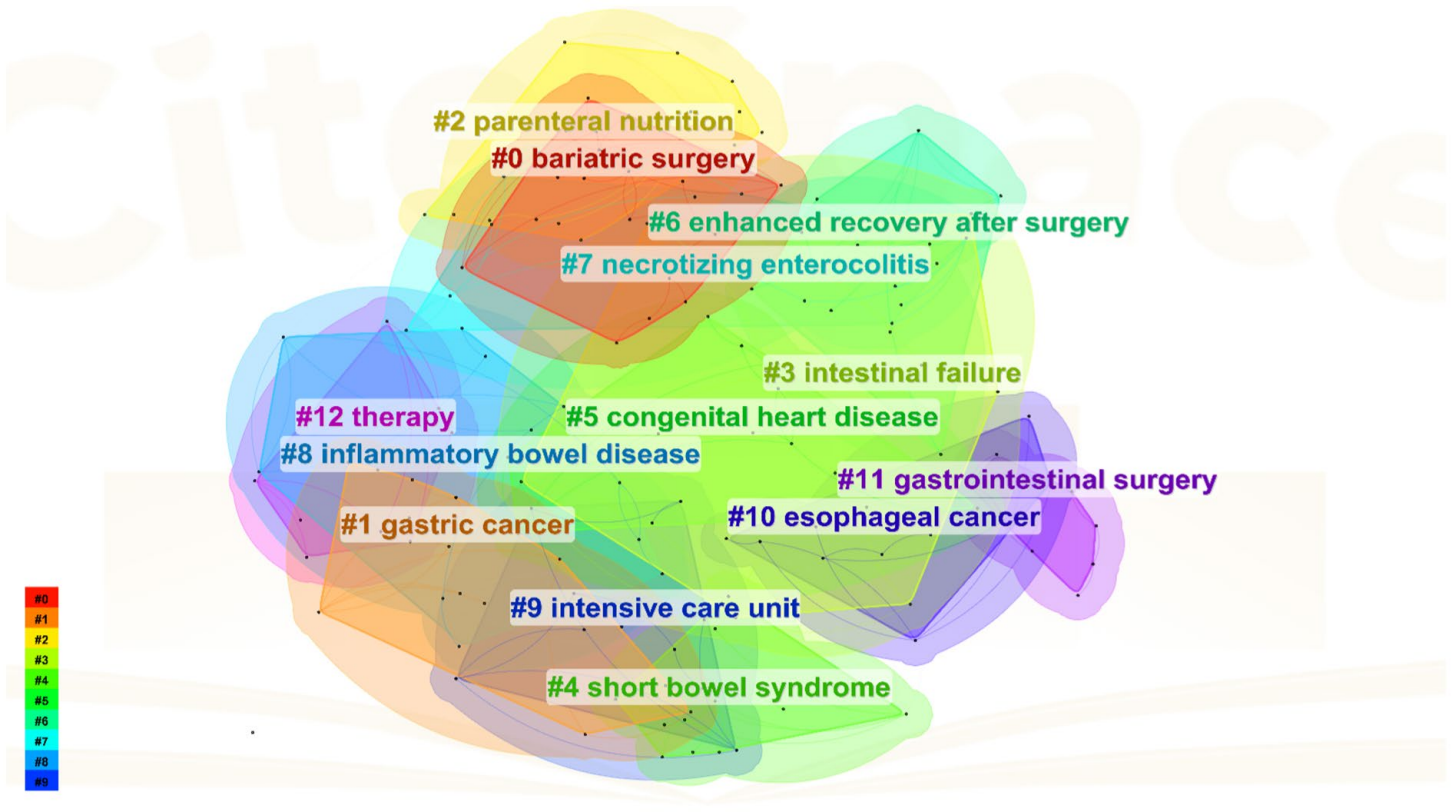
Cited cluster analysis showing the 13 main research areas.

The keyword clustering analysis synthesizes the co-occurrence network into clusters with specific, numerically assigned labels, shaped by a specialized algorithm. These labels are numerically assigned. The labels are numbered, with lower numbers indicating clusters containing more keywords, indicating a close connection among the keywords within each cluster. In this study’s keyword clustering map of this study, a *Q* value of 0.8315 indicates a substantial clustering structure, and an S value of 0.9697 implies highly convincing clustering results. The primary clusters identified include: #0 bariatric surgery, #1 gastric cancer, #2 parenteral nutrition, #3 intestinal failure, #4 short bowel syndrome, #5 congenital heart disease, #6 enhanced recovery after surgery etc. (Figure 8).

#### 3.7.3 Keyword timeline map

**Figure 9:**
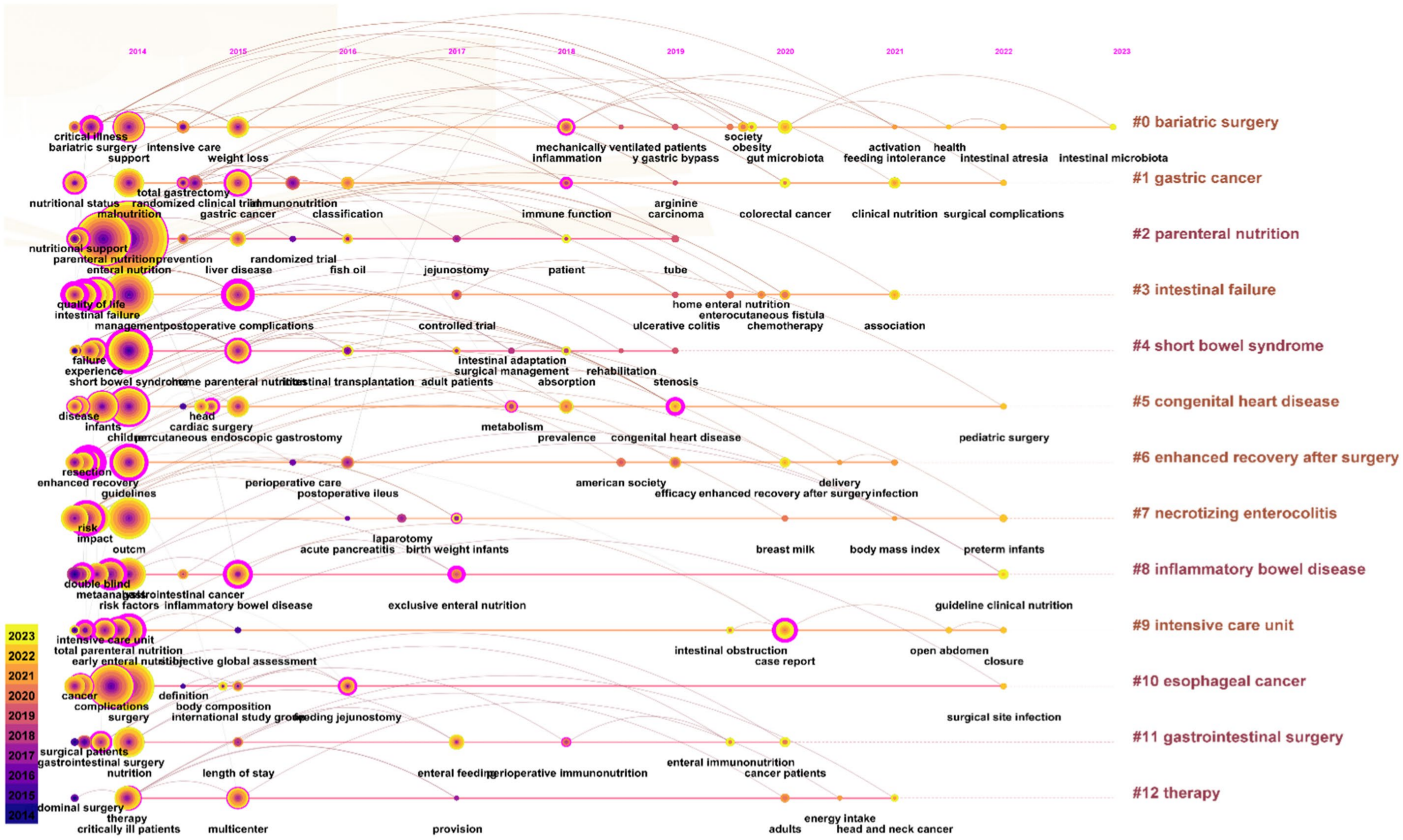
Time dynamic evolution of keywords in the PEN study.

A keyword timeline map is instrumental for tracing historical developments and projecting future trends within a field, dynamically illustrating the evolution and scope of knowledge. Figure 9 indicates that research emphasis in PEN has increasingly shifted towards such as intestinal microbiota, surgical complications, pediatric surgery, preterm infants, etc.

#### 3.7.4 Keywords with burst impact

**Figure 10:**
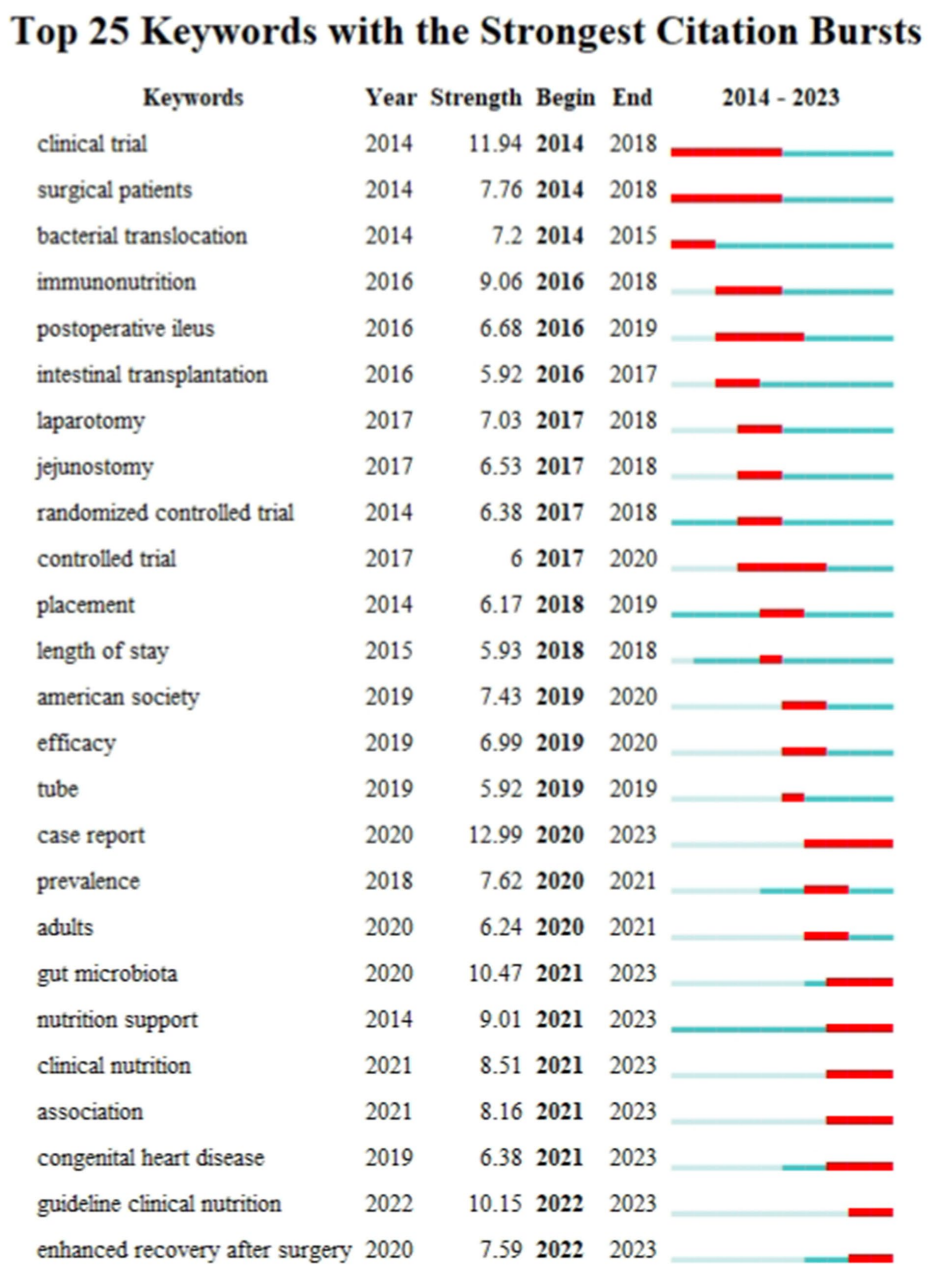
Top 25 keywords with the strongest citation bursts in the PEN study.

A keyword burst map was utilized to identify shifting focal points within the research domain, highlighting current and emerging research directions. Figure 10 shows the top 25 keywords with the strongest citation burst. Newly prominent keywords during the 2020–2023 period include “Gut microbiota,” “Nutrition support,” “Clinical nutrition,” “Association,” “Congenital heart disease,” “Guideline clinical nutrition,” and “Enhanced recovery after surgery.” These sustained bursts not only underscore the current research importance of these topics but also suggest potential future research directions.

## 4 Discussion

This study performed a visualization analysis using CiteSpace 6.3.1 (basic) on 3,768 documents published from 2014 to 2023, sourced from the WoSCC. The analysis covered temporal and geographical distribution, journal distribution, references, keyword hotspots, and frontier topics, leading to the conclusions.

### 4.1 General description

From 2014 to 2023, there was a consistent increase in the volume of literature, reflecting a rise in both the frequency and depth of research in this field. By 2023, publications more than doubled compared to 2014. This trend is likely due to the growing recognition of the importance of PEN. A significant peak in publications occurred in 2020, with 530 articles, likely driven to the global outbreak of COVID-19, which highlighted the link between malnutrition and infection in perioperative infection prevention and control. Consequently, it is necessary to enhance EN support, bolster patient immunity, and prevent worsening infections. Researchers and medical professionals have increasingly focused on perioperative management to improve treatment outcomes and recovery during the COVID-19 period. Factors such as medical policies, public attitudes, and economic progress, have introduced deviations between actual publication data and theoretical models. Based on the Figure 2, a future decline in publication volumes in PEN is anticipated. It is recommended that future studies analyze publication volumes over an extended period for a more accurate model.

As depicted in Figure 3, developed countries in Europe and America demonstrate broad and close collaborations. However, while countries like China and Japan produce a high volume of publications, they rarely engage in international collaborations. These countries should bolster their global research presence, perhaps through hosting international symposiums and participating in global research projects to address the current imbalance in worldwide research and development. Figure 4 highlights institutions such as Harvard University, the University System of Ohio, the University of Toronto, the University of California System, Assistance Publique Hopitaux Paris (APHP), Ohio State University, and Karolinska Institutet as prominent contributors in the PEN domain, noted for their extensive collaboration. Their success can be attributed to superior medical resources and robust financial support.

The analysis of journal co-citation indicates that the journals with high productivity cover five major disciplines: nutrition, pediatrics, surgery, internal medicine, and gastroenterology. This underscores the importance of multidisciplinary collaboration in PEN. Thus, future development in PEN is likely to be centered in these journals. These findings assist potential authors in identifying innovative research areas and selecting suitable journals for their publications.

### 4.2 Research hotspots in PEN

#### 4.2.1 Applications and challenges of PEN based on enhanced recovery after surgery (ERAS) for abdominal surgery patients

Enhanced recovery after surgery is grounded in evidence-based medicine, optimizing perioperative clinical pathways through multidisciplinary collaboration among surgery, anesthesia, nursing, and nutrition. It aims to alleviate patient stress responses, reduce complications, shorten hospital stays, and promote recovery. Poor preoperative nutrition can lead to missed optimal surgery times and stands as an independent risk factor for complications post-gastrointestinal surgery (21), closely associated with postoperative secretion of digestive fluids and surgical site infections (22). Therefore, standardized preoperative nutritional assessments are crucial for early postoperative EN. Currently, there is no unified standard for assessing or diagnosing malnutrition, thus it is recommended that medical staff flexibly use a variety of assessment tools for comprehensive consideration. The primary goal of prehabilitation is to make the patient “fit for ERAS” and at least to prevent further weight loss. ONS is the preferred strategy for preoperative nutritional prehabilitation (23). The American Society for Enhanced Recovery and Perioperative Quality states that preoperative daily protein intake should be more than 1.2 g/kg, and the length of preoperative nutritional supplementation should be determined based on the patient’s nutritional condition (24). Following ERAS principles, early postoperative EN has been proven to have a more direct effect on restoring gastrointestinal motility. A regression analysis (25) showed that the longer patients were restricted from an oral diet after surgery, the greater the time was to first bowel movement and the greater the postoperative complication rates (*P* < 0.0005). The ESPEN guideline recommended that EN should be initiated within 24 h after surgery, with patients undergoing lower gastrointestinal tract surgery starting EN within hours thereafter (17). Moreover, the safety of EN, particularly in patients with esophageal tumors undergoing radiotherapy and chemotherapy, requires enhanced safety evaluations and protections for early enteral nutrition post-esophageal cancer surgery. The nutritional requirements for EN vary among patients undergoing different abdominal surgeries, with the use of immunonutrition formulas in gastric cancer patients remaining controversial. A systematic review by Ma et al. (26) indicated that ω-3 polyunsaturated fatty acid significantly reduced postoperative infectious complication rates and shortened hospital stays and the duration of systemic inflammatory response syndrome. However, well-nourished patients (Nutrition Risk Screening 2002 < 3) with gastrointestinal cancer scheduled for major elective abdominal cancer surgery, did not benefit from dietary supplements containing ω-3 fatty acid taken 3 days before surgery (27). Patients undergoing bariatric surgery should supplement a comprehensive array of micronutrients and minerals (including folic acid, vitamin B12, vitamin D, thiamine, iron, zinc, copper, selenium, etc.) both preoperatively and postoperatively to counteract malabsorption following weight loss surgery (28). In patients with inflammatory bowel disease, exclusion enteral nutrition has shown promise as a preoperative optimization strategy for reducing complication in Crohn’s disease patients (29). However, no studies have assessed the use of exclusion enteral nutrition in patients with ulcerative colitis. In summary, clinicians should integrate individual situations of patients with standardized ERAS pathways to provide personalized nutritional support, thus enhancing the scientific and effective implementation of PEN.

#### 4.2.2 Prevention of enteral nutrition feeding intolerance in critically illness

Cancer patients often experience diminished appetite, reduced dietary intake, and weight loss, making them primary targets for EN. Patients with solid tumors, those unable to consume orally or achieve 60–75% of their targeted nutritional intake, and those with pre-cachexia or cachexia, all require EN (30). Critically ill patients, with a high incidence (2–75%) of ENFI, represent another focus area (31). These patients typically present with severe conditions, unstable hemodynamics, and varying levels of impairment to critical organs, leading to gastrointestinal dysfunction and often resulting in feeding intolerance (FI). When excessive enteral nutrition fluid accumulates in the digestive tract, it can cause aspiration pneumonia and extend the length of mechanical ventilation and ICU stays. Various EN delivery methods, like nasoenteric, nasogastric, and gastrojejunostomy tubes, require meticulous care. Therefore, vigilance against tube blockage, dislodgement, gastric content reflux, or aspiration during EN implementation is crucial to prevent adverse nursing events. However, the longer the duration of TF, the higher the risk of developing symptoms of tube feeding dependency. From a neurophysiological perspective, Pahsini et al. (32) discovered that particularly in premature infants in the NICU, the continuous and steady infusion of enteral nutrition keeps the body perpetually sated, leading to physiological or psychological anorexic behaviors during tube feeding phases. Thus, intermittent, personalized feeding strategies are recommended during this stage for precise control over nutrient intake.

#### 4.2.3 Prevention and management of perioperative enteral nutrition complications

As illustrated in Figures 5, 8, intestinal failure (IF) and short bowel syndrome (SBS) are burgeoning research focus in PEN. IF, defined as the gastrointestinal tract’s inability to sustain life without supplemental parenteral nutrition or intravenous fluids for at least 60 days (33). SBS often caused by extensive resections or bypasses of the small intestine, epitomizes chronic IF. It leads to a marked decrease in the intestines’ absorptive capacity, resulting in symptoms like diarrhea, acid–base imbalances, and compromised nutrient absorption and metabolism (34). It is reported that the incidence of SBS in adults undergoing intestinal resection is 15% (35), with 75% resulting from resections for colorectal cancer, and 25% from multiple consecutive intestinal segment resections. The ESPEN guideline recommended supplementing glutamine (Gln) and dietary fiber for patients with chronic IF when administering EN, as both nutrients enhance the intestinal mucosal barrier and positively influence gut microecology (36). Specifically, Gln reduces intestinal wall permeability and prevents translocation of gut flora, while dietary fiber stimulates growth and cell proliferation in the small intestine and colon mucosa. Moreover, individualized EN plans must be tailored to the specific characteristics and nutritional requirements of SBS patients, with regular assessments using clinical, anthropometric, and biochemical parameters to ensure regimen adequacy (37).

### 4.3 Frontier analysis in PEN

#### 4.3.1 Frontiers may focus on gut microbiota

The focus on gut microbiota gained prominence from 2020 to 2023, driven by its recognized role in perioperative nutrition and rehabilitation. According to Chowdhury et al.’s systematic review (38), perioperative use of probiotics or synbiotics significantly reduced the risk of postoperative infections following abdominal surgery and shortened overall hospital stays (*p* < 0.01), with synbiotics proving more beneficial than probiotics alone (*p* < 0.01). Therefore, probiotics or synbiotics can be used as safe and effective EN supplements after elective abdominal surgery. However, due to the heterogeneity of the included studies, it was not possible to ascertain which bacterial strains are most effective in reducing complications. Future research could employ high-throughput sequencing to investigate the relationship between gut microbiota diversity and postoperative complications, and through clinical trials, assess the effects of different types and doses of probiotics on patients’ gut microbiomes and postoperative recovery.

#### 4.3.2 Pay attention to the PEN management of congenital heart disease patients

It is crucial to explore how preoperative EN can improve the nutritional status of infants with congenital heart disease before surgery. Altered metabolic demands and compromised blood flow to the intestine in this population can lead to malnutrition, cellular hypoxia, insufficient energy intake, and poor oral motor skills (39). Mills et al. (39) have delineated the optimal time, methods, safety, and benefits of PEN for newborns with congenital heart disease based on best evidence practices. Teng et al. (40) conducted a retrospective cohort study among children born with complex congenital heart disease, undergoing biventricular repair before the age of four, between February 1999 and March 2009. Their finding suggested that early tube feeding was associated with decreased BMI during early childhood, indicating the potential need for prolonged nutritional monitoring and support even beyond the duration of tube feeding. These studies provide valuable insights for medical staff in optimizing PEN usage in children with congenital heart disease, ultimately enhancing surgical outcomes and quality of life.

## 5 Strengths and limitations

Employing bibliometric techniques, complemented by co-word analysis and literature review, this study has systematically examined PEN research since 2014. This paper outlines annual publication volumes, distribution by countries/regions, active institutions, co-cited references, core journals, research hotspots, and emerging frontiers, providing researchers with current and prospective insights into this field. Nonetheless, this study has the following limitations. Firstly, to meet the reference format requirements of the CiteSpace, we only searched the Web of Science Core Collection database and future studies could expand to include additional databases to enrich the data sources. Secondly, limiting the study to English language documents may inadvertently overlook substantial research contributions published in other languages. Thirdly, the reliance on CiteSpace and its inherent algorithms may have introduced certain biases in the results.

## 6 Conclusion

This study, utilizing CiteSpace, explored the research hotspots in PEN. Over the past decade, there has been significant advancements in this field, though the volume of future publications may diminish. There remains a development imbalance across various countries/regions and institutions. Evidence-based medical is increasingly preferred by researchers and clinicians, enhancing the scientification of clinical practices. Preoperative nutritional rehabilitation and early postoperative EN based on ERAS are currently prominent topics. Additionally, EN poses challenges during the perioperative period for various abdominal surgeries. Emerging trends, such as the gut microbiota and congenital heart disease patients, are poised to influence future research trajectories. The findings of this study provide a valuable reference for researchers and clinicians in the application and management of PEN.

## Data Availability

The original contributions presented in the study are included in the article/supplementary material, further inquiries can be directed to the corresponding author.
